# Economic burden on caregivers or parents with Down syndrome children—a systematic review protocol

**DOI:** 10.1186/s13643-022-02165-2

**Published:** 2023-01-06

**Authors:** Jyothi Shetty, Ankitha Shetty, Suneel C. Mundkur, Tantri Keerthi Dinesh, Prachi Pundir

**Affiliations:** 1 Department of Commerce, Manipal Academy of Higher Education, Manipal, Karnataka India; 2 Department of Paediatrics, Kasturba Medical College, Manipal, Karnataka India; 3 The George Institute for Global Health, New Delhi, India

**Keywords:** Economic burden, Financial burden, Out-of-pocket, Direct healthcare cost, Indirect cost, Caregivers, Parents

## Abstract

**Background:**

Financial burden is a common phenomenon, often noticed in the caregivers of children with Down syndrome. It echoes adverse effects on the caregiver’s mental and physical health. The economic burden covers direct healthcare costs, direct non-health-care costs, and indirect costs and is substantial for the family of a person with Down syndrome, as well as for society. Evidence, in this area, is necessary to reduce mental stress and promote financial well-being among caregivers.

**Methods:**

In this review, quantitative studies that assess the economic burden on caregivers of children with Down syndrome will be considered. We will perform a systematic literature search conducted from the year 2000 to 2022 on electronic databases CINAHL, EBSCO, EMBASE, PubMed, Scopus, Web of Science, and EconLit. An additional gray literature search will be carried out. Two researchers will independently conduct the screening and data extraction and assess the risk of bias.

**Discussions:**

The review attempts to methodically analyze the economic burden among caregivers of children with Down syndrome from the societal perspective and individual perspectives. The current study will provide an evidence base to researchers, academicians, and society in identifying need-based learning to caregivers, and the selection of appropriate therapies for children suffering from Down syndrome.

**Systematic review registration:**

PROSPERO CRD42021265312

**Supplementary Information:**

The online version contains supplementary material available at 10.1186/s13643-022-02165-2.

## Introduction

Despite the fact that there is a significant improvement in the knowledge of different genetic disorders, Down syndrome is the most common one leading to intellectual disability that still needs to be studied [1, 2]. People with Down syndrome suffer from chronic diseases such as congenital heart defects, hypothyroidism, immunological diseases, leukemia, and obstructive sleep apnea [3, 4]. India’s population is 1.3 billion [5] of which 26.8 million people are categorized as disabled, which accounts for 2.21% of the total population. As per the reports of the World Health Organization, the incidence of Down syndrome is found in 1 among every 1000–1100 children born alive [6]. According to the Down syndrome federation of India, about 30,000 children are born with Down syndrome, which is about 1 in every 800 to 1200 children born alive in India. In the last decade, the incidence of Down syndrome has increased by about 30%. The frequency of the increase in cases of Down syndrome highlights a need for resources required for this population [7]. Each year, approximately 3000 to 5000 children are born with this chromosome disorder. One of the vital public health issues worldwide, Down syndrome imposes a heavy burden on the family and society. However, there are minimal studies to assess the global burden of Down syndrome at present, to our knowledge. Individuals with Down syndrome can achieve optimal quality of life through parental care and support, medical guidance, and community-based support systems such as inclusive education at all levels. A family having a person with Down syndrome will have a profound demand on their available funds for the rest of their life [8]. Down syndrome has been studied in different parts of the world for several decades. In turn, the analysis of statistical data in combination with research studies demonstrates that this genetic disorder affects 1 person among 700 individuals. Researchers have opined that families of children with intellectual disabilities face a great amount of out-of-pocket costs [9]. Care is very much essential for children with Down syndrome, whereby this role is generally exercised by parents, where the actual anguish commences at diagnosis. This occurs due to the social meanings and beliefs of parents about disability. Caregivers of Down syndrome children encounter difficult challenges as they grow older including the development of dementia and depression [10]. The number of stress experienced by caregivers in the upbringing of the children can adversely affect the family’s quality of life [11]. Caregivers especially mothers of Down syndrome children take on the responsibilities of providing care and support [12, 13]. They will have the most influence on the personal health and wellness of a child with a disability, more so than any other individual or healthcare provider [14].

To maintain a high-quality of life, support is often needed from birth and includes early and intensive therapeutic services such as occupational and physical therapy as well as speech and motor therapies [15]. Apart from this caregivers’ experience financial stress that incurs from the costs of special education, medical and therapy appointments, childcare, and entertainment for their child with Down syndrome [16].

The financial burden of the family automatically elevates when one of the parents, most likely the mother tries to reduce working hours or step out of the job to take care of their disabled child [17].

A focus on the unmet needs of caregivers of children with Down syndrome is crucial to understanding how to improve current support services [18]. Regardless of the high costs, the majority of parents look out for early intervention strategies that help for child’s betterment suffering from Down syndrome. The costs are defined as “the value of the resources that are expended or forgone as a result of a health problem. It includes health sector costs (direct costs), the value of decreased or lost productivity by the patient (indirect costs), and the cost of pain and suffering (intangible costs)”. Like many other childhood disabilities, parents having a child with Down syndrome very often face difficulties in terms of time and money when compared with a neurologically typical child [19]. Down syndrome children need specialized childcare treatment which incurs heavy costs and has to be sustained for a longer period [20]. Due to the proliferating healthcare utilization associated with this condition, the economic costs related to Down syndrome are estimated to rank as one of the highest amidst intellectual disabilities [21].

Within the current literature, there is limited research focusing specifically on the support needs of caregivers of children with Down syndrome [22]. Substantial research efforts have been diverted toward the caregiver’s burden and quality of life (QOL) of children with Down syndrome [23]; on the contrary, less attention has been paid to the economic burden sustained by families affected by Down syndrome children. To date, only a small number of studies have investigated the support needs of caregivers of children with Down syndrome, and to our knowledge, a systematic review on the economic burden on family caregivers of children with Down syndrome has not yet been performed. The primary motive of this systematic review is to synthesize and evaluate the current literature regarding the economic burden on family caregivers of Down syndrome children with a profound principle of highlighting the gaps in the literature. The systematic review will also provide recommendations for policy and practice, and implications for research, based on the analysis of the economic data. The evidence from this review will inform government and non-government organizations and agencies globally, in planning, mobilizing the resources, and rolling out interventions for families and children with Down syndrome.

Even though there is growing concern among caregivers, literature shows that minimal research on the cost of illness has been published [24]. Much of the research has been carried out on the economic burden caregivers with cancer patients [25], Alzheimer’s disease [26], but not in Down syndrome. An urgent need to build a review of what is already known regarding caregivers’ economic burden in Down syndrome children is to be focused on the support of published contemporary research.

The present systematic review aims to determine the economic burden, in terms of direct and indirect costs incurred by caregivers of children with Down syndrome

## Methods/design

The protocol is reported following the requirements of Preferred Reporting Items for Systematic Reviews and Meta-Analysis Protocols (PRISMA-P) [27]. Standard methods shall be considered for data extraction, data synthesis, and meta-analysis. In addition, the Participants, Interventions, Comparisons, and Outcomes (PICO) strategy will be adapted as population, exposure, and outcome to suit the scope of the review.

### Inclusion criteria for study selection

#### Study design

The review will include observational studies such as cross-sectional studies and follow-up studies that focus on the economic burden of caregivers of Down syndrome children. Mixed-methods studies will be included (we will assess the quantitative component of the mixed-method study). Partial economic evaluation such as cost, cost of illness, and resource utilization analyses; and full economic evaluation such as cost-effectiveness, cost-utility, cost minimization, and cost-benefit analysis studies will be included. Cost data will be examined from full economic evaluations, but in case of the unavailability of cost data from such studies, the study will be excluded. We will include empirical literature as well as modeling studies of economic evaluation. The language will be restricted to English. Letters to editors and editorials, commentaries, conference proceedings, narrative reviews, systematic reviews, and case reports/series will be excluded.

#### Population

Participants are the caregivers/parents of people with all levels (mild, moderate, severe, and profound) of Down syndrome children of all age groups. Population covers all types of Down syndrome like Trisomy 21, translocation, and Mosaic Down syndrome.

#### Outcome measures

The primary outcomes will include any outcome related to economic burden in terms of direct and indirect costs incurred by caregivers of children with Down syndrome. This review will include studies publications that (a) have a focus on caregivers’ financial burden emphasizing their quality of life as well, (b) report empirical results and modeling studies about the economic burden of caregivers which encompasses direct and indirect costs with taking care of Down syndrome children, (c) are based in a special school environment, (d) are in English language, and (e) published between the year 2000 and 2022.

#### Search strategy

The literature search will be performed which will be conducted from the year 2000 to 2022 on CINAHL, EBSCO, EMBASE, PubMed, Scopus, Web of Science, and EconLit. Additional search will be conducted on Google Scholar, National Down Syndrome Society, Down syndrome Federation of India, Down Syndrome Association, and Down Syndrome education databases. A comprehensive search strategy using indexed descriptors and keywords will be developed. The keywords that will be used are “Down syndrome,” “trisomy 21,” “economic burden,” “economic cost,” “financial cost,” “financial burden,” “financial stress,” “direct cost,” “indirect cost,” “out-of-pocket cost,” “direct health care cost,” “caregivers,” “parents,” and “caretakers.” The initial search will be conducted in PubMed and will be translated for other databases. Reference lists of the included studies and previously published reviews will also be searched to get other relevant articles. An example of the search strategy for PubMed is provided in Table 1.

**Table 1:** Search strategy for PubMed

Strategy: #1 and #2 and #3		
	Concept	Query
#1	Economic burden	“Economic cost” or “economic burden” or “economic stress” or “financial cost” or “financial burden” or “financial stress” or “financial constraint” or “direct cost” or “indirect cost” or “out-of-pocket” or “healthcare cost” or “direct health care cost” or “direct non-healthcare cost” or “cost-of-illness” or “cost-effectiveness” or “cost-benefit”
#2	Population	“caregivers” or “parents” or “caretakers” or “mother” or “caretaker” or “father” or “families”
#3	Condition	“Down syndrome” or “trisomy 21” or intellectual disability” or “intellectual disabled” OR “intellectually disabled”

### Data collection and analysis

#### Study selection

The search results acquired from electronic database searches, and hand searches will be merged and deduplicated using reference management software (Zotero). All titles and abstracts will be examined by two researchers (JS and AS) independently against set inclusion/exclusion criteria to remove irrelevant articles. A senior reviewer (PP or SM) will be involved in resolving the disagreements on inclusion. The articles included for full-text screening will be retrieved and examined independently by two reviewers (JS and AS) whereby any disagreement will be discussed till consensus, and a final decision will be taken in discussion with a third reviewer (PP or SM). Detailed records of the outcomes at every stage, including all the rejected articles with reasons for rejection at the full-text stage, will be reported and depicted in PRISMA 2020 flow diagram [28]. The reasons for exclusion will be stated for the articles excluded at the full-text screening stage.

#### Data extraction

The studies included at the full-text stage will be carried forward for data extraction. Data extraction will be performed independently by two researchers (JS and AS) using the piloted data extraction form on Microsoft Excel. Disagreements will be discussed between two reviewers (JS and AS), and a third reviewer/senior reviewer (PP) will examine the extracted data and give a final decision. The following data will be extracted: (A) author and publication details, (B) country/region, (C) study objective, (D) methodology used for the study, (E) the demographic details of the participants and population sub-group, (F) cost incurred in currency reported in the study and United States dollar (USD) conversion, (G) statistical data, (H) other key findings, and (I) conclusion.

#### Dealing with missing data

In case of any missing data, the concerned author of the respective research paper will be contacted for further information. If there is a delay or no reply from the concerned author, the co-authors, or the corresponding author of the research study will decide upon inclusion/exclusion of the study in the systematic review.

#### Quality and risk of bias assessment

The observational studies (cross-sectional or other follow-up studies) will be assessed for quality using the New-Castle Ottawa scale [29]. Quality assessment of the eligible partial economic evaluation will be done by using the Consensus Health Economic Criteria (CHEC) list [30]. The full economic evaluation will be critically carried out using the Consolidated Health Economic Evaluation Reporting Standards (CHEERS) checklist [31].

#### Data analysis

We will present a table with the characteristics of included studies and report aggregate costs and sub-group costs for a country or population. The country-specific sub-group costs and year will be reported to reduce the bias that may be induced by currency value. The sub-group cost analysis will be carried out for different sub-groups of population (single or both parents, or grandparents or other caregivers).

For the cost data, the mean costs will be calculated from the included studies considering the homogeneity in the studies. A meta-analysis using a random-effects model will be performed if we come across homogenous data. Alternatively, or concomitantly, a narrative synthesis will be performed if we come across clinical, methodological, or statistical heterogeneity in methods or population of interest. Depending on the type of data, appropriate effect measures (such as mean, mean difference, odds ratio, and risk ratio) with a 95% confidence interval (CI) will be reported. Aggregate data will be used from the quantitative studies. The authors will try to conclude based on the type and quality of evidence available. The results will be subjected to a narrative summary or sorting in tables by subgroups, comparisons, and/or outcomes.

The study will include articles from various countries over the last two decades. To adjust for inflation and difference in currency exchange rates for US dollar, we will use the historical rate tables (from xe.com) to convert the currency rate to the year (last date) of data collection of study. We will use this historic rate (units per type of currency, specific to year of publication) as a multiplication factor for the USD rates. For data analysis, we will convert these rates to the current USD rates using XE currency converter. Additionally, we will report the adjusted converted rates along with the actual cost data given in the included study.

## Discussion

This will be the first systematic review to consolidate the economic burden on caregivers/parents of people with Down syndrome. Extensive and considerable search strategies and inclusion criteria will be contemplated in the present review, describing a systematic review of the obtainable evidence where detailed retrieval strategies are being formulated, and there are no possibilities of including unpublished trials. There will be no restriction concerning the geographical region and will consider studies globally, and we will not be including case studies and qualitative studies. We anticipate that the studies on this topic may be scattered in terms of geographic location or type of population or study design.

The review attempts to methodically analyze the economic burden of children suffering from Down syndrome from the societal perspective and also the perspective of caregivers of such children. The research gap will be highlighted in terms of the type of research, geographic location, and type of population in the studies. The current study will provide an evidence base to identify the financial burden and plan evidence-informed interventions for children suffering from Down syndrome. The study will provide insight into the challenges of families of people with disabilities and draw the attention of the policymakers, government and non-government organizations, health care professionals, researchers, academicians, and civil society towards this issue. Recognizing and taking steps towards addressing these challenges will reduce mental stress and promote financial well-being among caregivers of children with Down syndrome.

## Supplementary Information

**Additional file 1.**

## Data Availability

Not applicable
